# Immediate Effect of a Yoga Breathing Practice on Attention and Anxiety in Pre-Teen Children

**DOI:** 10.3390/children6070084

**Published:** 2019-07-22

**Authors:** Shirley Telles, Ram Kumar Gupta, Kumar Gandharva, Babita Vishwakarma, Niranjan Kala, Acharya Balkrishna

**Affiliations:** Patanjali Research Foundation, PatanjaliYogpeeth, Haridwar 249405, Uttarakhand, India

**Keywords:** pre-teen children, attention, anxiety, yoga breathing, yoga based breath awareness, sitting quietly

## Abstract

Pre-teen children face stressors related to their transition from childhood to adolescence, with a simultaneous increase in academic pressure. The present study compared the immediate effects of 18 min of (i) high frequency yoga breathing with (ii) yoga-based breath awareness and (iii) sitting quietly, on (a) attention and (b) anxiety, in 61 pre-teen children (aged between 11 and 12 years; 25 girls). Attention was assessed using a six letter cancellation task and Spielberger’s State Trait Anxiety Inventory STAI-S was used to measure anxiety before and after the three practices, practiced on separate days. Repeated measures ANOVA, followed by Bonferroni adjusted *post-hoc* analyses showed an increase in total attempts and net scores after high frequency yoga breathing (*p* < 0.05), while wrong attempts increased after yoga based breath awareness (*p* < 0.05). Anxiety decreased comparably after all three interventions. The 25 girls in the group had the same trend of results as the whole group with respect to the attention-based cancellation task, while boys showed no, how since change. For both girls and boys, anxiety decreased after all three 18min interventions. The results suggest that high frequency yoga breathing could be a short, useful school based practice to improve attention and reduce anxiety.

## 1. Introduction

Pre-teen children are at a stage of both promise and challenges, as they transition from childhood to being teenagers. Some of the challenges include an increase in academic pressure, a greater involvement in family and social dynamics, adjusting to changing social and behavioral demands of their peer group, and sometimes a growing difference in values and opinion with their parents and guardians [1]. Along with these stressors, in India pre-teens face a definite increase in academic load as they begin to prepare to answer an important, competitive, and career-determining school leaving examination in the next two to three years [2]. Academic pressure is a particularly important factor in those pre-teens the world over, who see education as a way to improve their own and their family’s social and economic prospects [3].

Researchers from medicine and public health disciplines have assessed the usefulness and benefits of yoga for children in school settings [4]. The benefits included improved psychological well-being, reduced rates of being overweight or obese, children being calmer and more attentive, more at ease, and completing assigned tasks successfully. In a separate study, yoga breathing techniques were introduced in a suburban, private, English medium school in north India [5]. Two sections of the third grade (children aged 7–8 years), with approximately 50 students in each section were selected. One section received yoga breathing (i.e., deep breathing and alternate nostril yoga breathing) as a 5min intervention every school day for the academic year. Scores obtained in Math and languages (English and Hindi) were noted at the beginning and end of the year. In the group who practiced yoga breathing there was a statistically significant increase in cumulative scores. Though the effect size was small (eta squared = 0.108) the teachers reported other benefits such as better attention, improved classroom behavior, and active participation.

The neurocognitive effects of yoga breathing were further discussed in a narrative review, which searched PubMed, PubMed Central, and IndMed for citations with ‘Pranayama’ and ‘Yogic breathing’ as the search words [6]. In the review, 68 studies reported the effects of yoga breathing alone and met other inclusion criteria, and hence were included. The studies were categorized and one sub-category was neurocognitive assessments. The yoga breathing techniques discussed included those which varied the rate of breathing, the depth of breathing, the nostril(s) breathed through, and where exhalation was associated with specific sounds. The details of these yoga breathing practices as well as others mentioned in this study are provided in Table 1.

The neurocognitive assessments included electrophysiological variables (i.e., EEG, evoked potentials, and event related potentials), spatial and verbal memory tasks, auditory and visual reaction time, the Purdue pegboard task, and substitution and cancellation tasks. The authors concluded that pranayama or yoga breathing influenced neurocognitive abilities positively and that some practices were also useful in a clinical setting.

Among yoga breathing techniques the effects of those which influenced the rate of breathing were compared in young adults aged 18–25 years [7]. Into three groups, 84 participants were randomized as (i) fast yoga breathing (rate not specified), (ii) slow yoga breathing (rate not specified) and (iii) a control group. Participants practiced the respective yoga breathing techniques for 35 min, thrice a week for 12 weeks. Assessments included cancellation tasks, trail making tests, digit span forward and backward, and reaction time (auditory and visual). With both fast and slow yoga breathing, performance in cancellation tasks, trail making tests, digit span forward, and auditory and visual reaction time improved, with a greater magnitude of improvement in the group who practiced fast yoga breathing.

Most of the studies described above assessed the effects of yoga breathing practiced for a number of days or weeks. However there are also acute or immediate effects of yoga breathing, which occur directly after the practice. Examples of the immediate effects of yoga breathing studied in children are discussed here. In 60 adolescents (mean age 14.6 years, 38 males), 45 min of yoga bumble bee breathing reduced the heart rate and blood pressure [9]. Yoga breathing with an increased depth of breathing (called bellows breath), when practiced as nine rounds, where one round was one breath cycle, reduced the auditory and visual reaction time significantly in 22 healthy school boys (mean age 14.5 years) [10]. A shorter reaction time suggested that participants responded sooner.

Similarly, the immediate effects of short durations of yoga breathing have been assessed in adults. Into two groups, 94 persons were assigned to(i) high frequency yoga breathing (mean age 39.3 years; 28 females; breath frequency 1.0 Hz) and (ii) breath awareness (mean age 39.8 years; 26 females) [8]. The O’Connor finger dexterity task and a shape and size discrimination task were administered to all participants just before and after 10 min of high frequency yoga breathing or breath awareness. There were improvements in the way participants performed both tasks, though the magnitude of change was greater after high frequency yoga breathing. These tasks require tactile discrimination, fine motor skills, and eye hand co-ordination; hence the results suggest these abilities improved to a greater extent with 10 min of high frequency yoga breathing. A similar trend was seen with an assessment of the degree of optical illusion perceived, which is a measure of visual discrimination, in 30 adult males (mean age 26.9 years), where a greater degree of improvement followed 15 min of high frequency yoga breathing at 1.0 Hz compared to breath awareness [11].

High frequency yoga breathing practiced at breath frequencies between 1.0 and 2.0 Hz improved the performance scores in an attention-based cancellation task as an immediate effect of practice across different age groups [12]. The cancellation task requires the ability to focus attention, shift attention, as well as other abilities such as visual scanning and psychomotor speed [13]. There was a 56.1 percent reduction in total errors in the cancellation task in medical students (*n* = 46, age range 18–23 years); a 32.5 percent improvement in the net scores of middle aged adults (*n* = 48, age range 30–59 years) and 16.4 percent in older adults (*n* = 16, over the age of 60 years) [12]. High frequency yoga breathing has not been specifically tested as a classroom based intervention in pre-teen children.

Hence the present study was designed to assess performance in an attention based cancellation task immediately before and after 18 min of high frequency yoga breathing in 61 pre-teen children, compared to sessions of yoga based breath awareness or quiet rest of equal duration, in a random sequence, on separate days.

## 2. Materials and Methods

### 2.1. Participants

There were 61 pre-teen children with ages ranging from 11 to 12 years (group mean age (SD), 11.2 (0.5) years) recruited for the study from a residential school in India. They were students of grade 6 and 7 and there were 25 girls. The sample size was not calculated prior to the trial. However, the power was calculated from STAI-S scores, which were significantly different between states in the repeated measures ANOVA and in *post-hoc* analysis comparing post values with pre in the high frequency yoga breathing (HFYB) session. With the sample size of 61, and the Cohen’s d of 0.49 (medium), the power obtained was 0.99 [14]. The children were recruited based on the following inclusion criteria: (i) Apparently normal health based on a routine case history and medical examination; (ii) aged between 11 and 12 years, both genders; (iii) staying in a residential school for at least 2 years, with bi-annual home visits; (iv) at least one years’ experience (with a frequency of 26 days/month) of yoga practice including high frequency yoga breathing (v) right hand dominant based on Edinburgh handedness inventory [15] and (vi) willingness to take part in the study. The exclusion criteria were: (i) Girls who attained menarche or boys who reached puberty, (ii) presence of any physical or mental illness and (iii) taking any medication.

The school was located in north India. Admission was competitive and students answered an exam and their parents were spoken to, to understand the family background before admission. Boys and girls are housed in separate accommodation but have classes together. The parents are allowed to visit the children 3 to 5 times a year and the children get holidays to go home in summer and winter for a total of two months in a year. There are 4 house-persons, who stay with the children and one class teacher per class. There are 40 to 50 students in a class. The students have activities starting from 04.30 h to 21.30 h, which were supervised. These activities included a 50 min yoga class in the morning, universal prayer, regular classes, games (this included team and individual games as well as competitive and non-competitive games) for 75 min, self-study for 60 min, and time for meals. The remaining time was for their individual activities.

The study was approved by the institution’s ethical committee (approval number: YRD-018/018-19). Signed informed consent was taken from the principal of the school. The principal informed the parents of the students about the study and their oral approval was also taken.

### 2.2. Study Design

The children were randomly assigned to 3 groups, i.e., group A, group B, and group C using an online random sequence generator [16]. The procedure was as follows: Children were given serial numbers from 1 to 61 independent of their name, order of enrolment, or any other factor. Using an online random generator, 61 random numbers were generated [16]. These random numbers were noted beside the serial number assigned to each child. The random numbers were then written on identical slips of paper and folded in a similar manner. The slips were mixed and a person who was unaware of the study design put the slips one at a time into three boxes labelled A, B, and C. The letters A, B, and C represented group A, group B and group C respectively.

After allocation of the children to three groups, the online randomizer [16] was used to assign the sequence of the three interventions to the three groups for three days. As a result, group A practiced HFYB, BAW, and QS on day 1, day 2, and day 3 respectively; group B practiced BAW, HFYB and QS on day 1, day 2, and day 3 respectively and group C practiced QS, BAW, and HFYB on day 1, day 2, and day 3, respectively. Assessments of SLCT and STAI were taken before and after each session. The data were taken in November, 2018.

### 2.3. Assessments

#### 2.3.1. Six Letter Cancellation Test (SLCT)

This is a standardized paper-and-pencil test, which is used to assess selective attention [12,13]. The SLCT consists of an A4 size sheet of paper in which letters of the English alphabet are randomly arranged in a 22 × 14 matrix. At the top of the test sheet there were6 target letters; these 6 letters were interspersed with non-target letters in the work sheet below. Participants were instructed to spot the 6 target letters in the work sheet and to cancel them with a slash (/). Cancellation of non-target letters was counted as errors and the total time given to complete the test was 90 s. The net score was calculated by subtracting wrongly cancelled letters from the total letters cancelled.

#### 2.3.2. Spielberger’s State Trait Anxiety Inventory – State (STAI-S)

The STAI-S is a standardized 20 item inventory, which assesses the anxiety, i.e., the anxiety at the moment of testing [17]. The 20 items are sentences which describe the feelings of a person which can be assessed on a 4-point (i.e., not at all, somewhat, moderately so, and very much so) Likert scale. The participants had to choose one out of the 4 options provided for each item. This scale was not culture sensitive and had been standardized for use in an Asian population (of which 6.5% was an Indian population) with test-retest reliability (r = 0.60–0.94) and Cronbach’s alpha score of 0.86 [17].

#### 2.3.3. Visual Analog Scales (VAS)

Visual analog scales were used to self-rate the quality of practice after each session. Each scale was a 10 cm horizontal line that allowed participants to self-rate the quality of practice on a scale of 0 (worst possible) to 10 (excellent). The participants were told to put a mark on the line to describe how well they were able to follow the instructions during the practice of HFYB, BAW, or QS.

### 2.4. Interventions

There were 3 interventions which each group practiced in 3 days in random order. These were (i) high frequency yoga breathing or HFYB, (ii) breath awareness (BAW), and (iii) quiet sitting (QS).

#### 2.4.1. High Frequency Yoga Breathing (HFYB)

HFYB consisted of rapid breathing with forceful exhalation contracting the anterior abdominal muscles, at a rate of 1.0 Hz (60 breaths per minute). Children were instructed to avoid using excessive force. During the practice the volunteers checked that the children appeared relaxed, especially their facial expression, shoulders, and trunk. The children were instructed to sit cross-legged with their eyes closed and spine and neck erect and aligned during HFYB. During this practice children were instructed to be aware of the passage of air through the nasal and respiratory passages, while also being aware of all the movements during the practice.

There were two teachers of each gender appointed by the school to teach yoga. Each of them had a minimum of 2 years training in yoga, both theory and practice. They were familiar with the contraindications to practice high frequency yoga breathing, i.e., a history of febrile convulsions or seizures due to any cause, recent surgery or injury to the thorax or abdomen, and any breathing disorder in the children, which would make it difficult to carry out the practice. The yoga teachers were also aware of the precautions they needed to observe i.e., check that the children were not hyperventilating, over-breathing, or fatigued.

#### 2.4.2. Breath Awareness (BAW)

BAW consisted of being aware of the movement of air in the nasal and respiratory passages without manipulating it. The children were asked to sit cross-legged with their eyes closed and neck and spine erect and aligned, and attention directed to awareness of the breath. Breath awareness was added as an interventional control because being aware of one’s breath is an essential part of yoga breathing. Hence this is yoga-based breath awareness, which for the sake of convenience is referred to as ‘breath awareness’.

#### 2.4.3. Quiet Sitting (QS)

QS was the control session in which participants were instructed to sit quietly with closed eyes and their neck and spine erect and aligned. They were instructed to allow their thoughts to wander without attempting to regulate or direct them.

**Figure 1:**
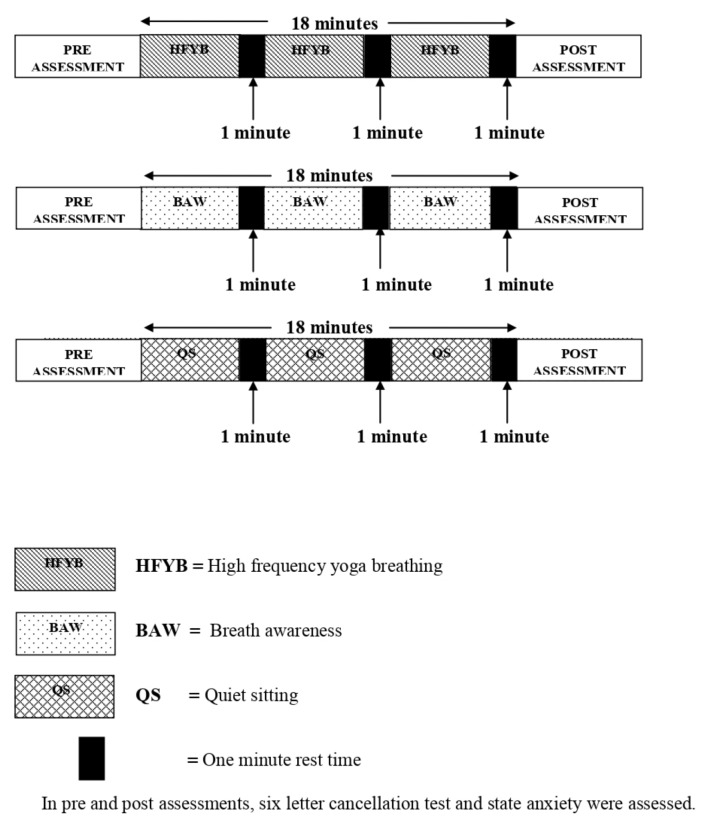
Schematic representation of the study design. This is a schematic representation of the study design. The interventions are as follows: The hatched rectangle indicates high frequency yoga breathing (HFYB), the stippled rectangle indicates breath awareness (BAW), the cross hatched rectangle indicates quiet sitting (QS) and the smaller darkened rectangles indicate rest periods of one minute duration.

The total time for each intervention was 18 min, which consisted of 3.5 min epochs of the respective practice followed by 1 min of rest after each epoch. The SLCT and STAI-S were assessed at the beginning and end of the sessions and the VAS was assessed at the end of each session. Figure 1 represents the study design schematically.

### 2.5. Data Analyses

Data analyses were done using SPSS (24.0). The data obtained before and after the 3 sessions were compared using a repeated measures analysis of variance (RM-ANOVA) followed by Bonferroni adjusted *post-hoc* analyses. The RM-ANOVA had 2 within-subjects’ factors i.e., states, with 2 levels (pre and post), and sessions, with 3 levels (HFYB, BAW and QS). The data were analyzed for the group as a whole, as well as for boys and girls separately.

Parametric tests (i.e., repeated measures ANOVA followed by multiple *post-hoc* tests) were used since with sample sizes greater than 30 whether the data were normally distributed (or not) does not impact accuracy [18].

## 3. Results

The group mean values (SD) for SLCT, STAI-S, and VAS are given in Table 2. ANOVA values for SLCT and STAI-S are given in Table 3.

### 3.1. Repeated Measures Analyses of Variance (RM-ANOVA)

In the whole group, the STAI-S scores differed between States (F = 39.606, df = 1,60, *p* < 0.001). A significant interaction between Sessions × States was noted in net scores of SLCT (F= 3.766, df = 2,120, *p* < 0.05) suggesting interdependence of the two factors [19]. The STAI-S scores differed between States for boys (F = 25.420, df = 1,35, *p* < 0.001) as well as for girls (F = 14.595, df = 1,24, *p* < 0.01). There was a significant interaction between Sessions × States for the total attempted scores (F = 5.791, df = 2, 48, *p* < 0.01) and net scores (F = 6.817, df = 2,48, *p* < 0.01) in girls alone.

### 3.2. Post-Hoc Analyses

For the whole group, there was a significant increase in the total attempted scores (*p* < 0.05; 95% CI = −4.75, −0.34) and net scores (*p* < 0.05; 95% CI = −4.47, −0.15) of SLCT after HFYB, while there was a significant increase in wrong attempts after BAW (*p* < 0.05; 95% CI = −0.97, −0.02). The Anxiety decreased significantly after all three sessions i.e., HFYB (*p* < 0.001; 95% CI = 3.09, 7.17), BAW (*p* < 0.001; 95% CI = 2.59, 6.82), and QS (*p* < 0.001; 95% CI = 2.73, 6.58). The average (SD) visual analog scale scores for HFYB, BAW, and QS were 8.4 (1.8), 8.3 (1.3) and 8.4 (1.6), respectively.

For boys alone, no changes were seen in the scores of SLCT after any of the three sessions; however the anxiety was decreased after all three sessions i.e., HFYB (*p* < 0.001; 95% CI = 3.10, 884), BAW (*p* < 0.01; 95% CI = 1.37, 6.90) and QS (*p* < 0.01; 95% CI = 1.39, 5.78). The average (SD) visual analog scale scores were 8.8 (1.8), 8.6 (1.3), and 8.7 (1.3) for HFYB, BAW and QS respectively for boys.

For girls alone, the total attempted scores (*p* < 0.05; 95% CI = −8.22, −0.58) and the net score (*p* < 0.05; 95% CI = −8.22,−0.58) of SLCT were significantly increased after HFYB. After BAW there was an increase in wrong attempts (*p* < 0.05; 95% CI = −1.83, −0.13) and a decrease in net scores (*p* < 0.05; 95% CI = 0.53, 5.71). The anxiety was decreased after all three sessions in girls also [(HFYB = *p* < 0.05; 95% CI = 0.96, 6.88), (BAW = *p* < 0.01; 95% CI = 2.01, 9.03), (QS = *p* < 0.01; CI = 2.60, 9.80)]. The average (SD) visual analog scale scores for HFYB, BAW and QS were 7.8 (1.2), 7.8 (1.7), and 7.8 (1.6), respectively, for girls.

## 4. Discussion

The study was intended to determine whether an 18 min school-based intervention would increase performance in an attention task and decrease anxiety. In 61 pre-teen children the level of anxiety decreased comparably immediately after 18 min of high frequency yoga breathing, breath awareness, and sitting quietly at rest. The performance in an attention-based, cancellation task improved after yoga breathing alone.

The decrease in anxiety after all three interventions may be due to some common as well as some distinct factors. Yoga breathing has been associated with reduced anxiety, which may be due to multiple factors [20,21,22]. Similarly breath awareness and mindfulness are widely known to reduce anxiety though different mechanisms. During breath awareness and mindfulness the mental state is focused on the breath and is less likely to be diverted to stressful thoughts. This benefit of mindful-awareness has also been shown in school settings [23]. Sitting quietly at rest may have induced a quiet meditative state in these pre-teen children who already had 1–2years’ experience of yoga, since an earlier study in adult yoga practitioners showed that persons with experience in yoga have lower levels of arousal demonstrated by lower basal metabolic rates at rest compared to non-yoga practitioners [24]. Sitting in silence has been shown to be stress reducing as an in-school intervention, practiced as quiet time [23].

Breath awareness in the present study cannot be considered the same or as effective as mindfulness meditation or mindful breath awareness. Breath awareness is a part of yoga breathing which may be as effective to reduce anxiety as sitting quietly with random thinking. However yoga-based breath awareness and mindful breath awareness can be expected to differ, as mindful breath awareness involvespaying attention to experiences as they occur along with maintaining a non-judgmental and accepting mental state, whilestable attention and associated cognitive processes arehighlighted [25]. The children in the present trial were trained in yoga breathing and in breath awareness as a part of yoga practice.

The cancellation task used in the present study, chiefly assesses the ability to focus and shift attention but also assesses short term recall (of the test letters), as well as visual scanning and motor performance [13]. In 107 adults (age range 18–30 years), active inhalation improved facial expression recognition and object recall [26]. Since high frequency yoga breathing involves exhaling actively and passively inhaling through the nose at an increased rate, this breathing practice may directly influence recognition and recall. In earlier studies on adults the immediate effect of high yoga breathing was an improvement in visual perception [11]. This ability may also have partially accounted for the improvement in the letter cancellation task performance in the present study. The present results suggest that yoga breath awareness, which does not involve breath manipulation is less efficient compared to yoga breathing with respect to the attention-based cancellation task, since an increase in wrong attempts in the group as a whole occurred after breath awareness.

The 25 girls in the study showed the same results as the whole group, i.e., better performance in the attention task following high frequency yoga breathing, increased wrong attempts after breath awareness and reduced anxiety after all three interventions. The boys showed a decrease in anxiety after all three interventions, with no increase in the attention task performance after any of them. This gender-difference may be explained on the basis of another study as follows. The differential effect of gender on the degree of engagement and the degree of effectiveness of a mindfulness program was compared in male and female adolescents [27]. Females were more engaged than males in the class and also reported less stress post-intervention. Despite the small sample size (10 female and 5 male adolescents), the results of the study may also explain the present gender differences following yoga breathing in which girls alone showed an increase in total attempts and net scores after yoga breathing, with increased wrong attempts after breath awareness. Apart from this, normative data for performance in the six letter cancellation task were obtained in India, in 819 school students between 9 and 16 years of age (mean age 12.1 years) [28]. The children had normal health and were proficient in English. The authors reported that girls had higher scores than boys but did not provide a possible explanation for the difference. However, given that girls score better than boys in the cancellation task this advantage may be further enhanced by an intervention, in this case, high frequency yoga breathing. Finally, the program for International Student Assessment, which is a triennial international survey has observed that even though several countries have been able to close gender gaps in learning outcomes—boys and girls differ in their attitudes towards learning—their behavior in school, how they choose to spend their leisure time, and the confidence they have (or do not have) in their own abilities as students [29,30]. These factors may have also been responsible for the gender-difference in the present study.

Since the present study aimed at assessing a yoga breathing intervention for use in a school setting, another study which attempted a similar exercise is discussed here. In this earlier study two yoga breathing practices were introduced for 5 min each school day for a year [5]. This was in an English medium school near New Delhi. Two sections of the third grade, where students are between 7 and 8 years of age were selected. One section (*n* = 54) was assigned the yoga intervention while the other section (*n* = 51) acted as the control. The scores in Math and languages (English and Hindi) were noted in both groups at the start and end of a year. The yoga group had a significantly higher cumulative gain in academic scores compared to the control group. The teachers also reported other benefits (e.g., an increase in attention span, more active participation in the class room) in the yoga group. The findings are limited by the fact that the children were not randomly assigned to the two interventions and the additional practice could have been a source of motivation to the yoga group, while the control group may have been less motivated in the absence of this additional attention [31].

Unlike the present study the teachers in the study cited above were trained for a week before introducing the yoga program in the classroom (whereas in the present study yoga teachers had a two year post graduate degree in yoga). The students also took a shorter time, as they were trained in one month (which included many holidays, as mentioned by the authors) [5]. The main reason for this could be the difference in yoga breathing practices. In the earlier study [5], children were taught deep breathing (which involved deep nasal inhalation and exhalation) and alternate nostril breathing (which involves inhalation and exhalation through left and right nostrils alternately). High frequency yoga breathing is appropriate for pre-teens and has been described to take approximately two weeks for beginners to reach a breath frequency of 22 breaths per minute if they started with 11 breaths per minute, after which participants are asked to increase the rate by 11 breaths per minute every two weeks [32]. Hence for novices to reach a breath rate of 55–60 breaths per minute (which was the breath rate practiced by the pre-teen participants in the present study), the time required would be 8–10 weeks.

In the present study the finding that 18 min of high frequency yoga breathing are beneficial, is limited by factors such as (i) the study was conducted in a residential school, which helped regulate experimental conditions but may not be entirely the same for pre-teens living at home, (ii) the children had already been practicing yoga for one to two years (iii) there were no assessments which would have allowed a better understanding of the mechanisms involved, and (iv) the 18 min yoga breathing intervention was not tested in novices to yoga.

## 5. Conclusions

There were 61 pre-teen children who showed an immediate response to 18 min of high frequency yoga breathing, breath awareness, and quiet sitting in terms of reduced anxiety. Following high frequency yoga breathing they obtained better scores in an attention-based cancellation task. In contrast on another day the same children showed an increase in errors after yoga-based breath awareness.

School curricula are very demanding. Fitting in a stress reduction practice is challenging. The present results suggest that high frequency yoga breathing if correctly taught can improve the ability to focus and shift attention as an immediate effect, while reducing anxiety. Given the immediate effects of the 18 min practice the intervention could be introduced at the start of the school day. Sitting quietly and yoga based breath awareness were also useful to reduce anxiety and could be considered as add-on practices to yoga breathing.

## Figures and Tables

**Table 1:** A brief description of the yoga breathing practices mentioned in this study.

No.	Aspect of Breathing Which Was Modified		Name of the Yoga Breathing Practice		Method
			Sanskrit	English	
1.	Rate	Fast yoga breathing	*Kapalabhati*	High frequency yoga breathing	Rapid nasal breathing with forceful exhalation by contracting the anterior abdominal muscles, followed by passive inhalation at a rate of 60 to 120 breaths/min [7,8].
			*Kukkuriya*	Dog pant like yoga breathing	After sitting in the diamond pose (*vajrasana*) with the palms on the ground in front; rapid breathing through the mouth is practiced with the tongue kept fully out of the mouth [7].
			*Bhastrika*	Bellows type yoga breathing	Deep inhalation followed by complete exhalation, with an increase in breath rate [7].
		Slowyoga breathing	*Nadishodhana*	Alternate nostril yoga breathing with breath-holding	The practice begins with exhalation from the left nostril, followed by inhalation from the nostril (while the right nostril is gently occluded with pressure from the fingers), then exhalation from the right nostril (occluding the left), inhalation from the right, followed by exhalation from the left nostril completing the cycle. This practice may include a period of breath-holding [7].
			*Pranav*	Om repetition with breathing	Practiced in four parts: (i)Lower chest breathing with the sound of AAA, (ii) mid-chest breathing with the sound of UUU, (iii) upper chest breathing with the sound of MMM and (iv) union of the earlier three parts with the sound of AAA, UUU and MMM. Exhalation lasts for duration of 2 to 3 times the duration of the inhalation [7].
			*Savitri*	Breathing related to the sun	Slow, deep, and rhythmic breathing with inhalation: Inhalation breath holding: Exhalation: Exhalation breath holding: In a ratio of 2:1:2:1 [7].
2.	Nostril breathed through		*Anulom-Vilom*	Alternate nostril yoga breathing	The practice begins with exhalation from the left nostril, followed by inhalation from the nostril (while the right nostril is gently occluded with pressure from the fingers), then exhalation from the right nostril (occluding the left), inhalation from the right, followed by exhalation from the left nostril completing the cycle. This practice does not include breath-holding [5].
3.	Depth		*Bhastrika*	Bellows type yoga breathing	Deep inhalation followed by complete exhalation, with an increase in breath rate [7].
4.	Exhalation with a sound		*Bhramari*	Bumble bee yoga breathing	Deep inhalation followed by prolonged exhalation along with a humming sound like a bumble bee [9].

**Table 2:** Mean values (SD) of the SLCT and anxiety pre and post HFYB, BAW, and QS sessions.

Whole Group (*n* = 61)												
Variables	HFYB				BAW				QS			
	PreMean (SD)	PostMean (SD)	Cohen’s d	*p*-Value	PreMean (SD)	PostMean (SD)	Cohen’s d	*p*-Value	PreMean (SD)	PostMean (SD)	Cohen’s d	*p*-Value
**TA**	27.15 (7.40)	29.69 (10.47)	0.280	0.024	28.14 (8.43)	27.38 (8.65)	0.089	0.351	27.91 (7.27)	28.72 (8.73)	0.101	0.394
**WA**	0.59 (1.20)	0.81 (1.91)	0.138	0.391	0.44 (0.89)	0.93 (1.93)	0.326	0.043	0.48 (1.02)	0.85 (2.50)	0.194	0.275
**NA**	26.55 (7.32)	28.86 (10.33)	0.258	0.036	27.70 (8.34)	26.44 (8.58)	0.148	0.134	27.49 (7.41)	27.87 (8.45)	0.048	0.623
**STAI-S**	37.59 (10.51)	32.46 (10.10)	0.498	<0.001	37.15 (10.53)	32.44 (9.93)	0.460	<0.001	36.63 (9.98)	31.98 (10.33)	0.458	<0.001
**Girls (*n* = 25)**												
**TA**	27.84 (8.10)	32.24 (12.20)	0.424	0.026	30.76 (8.23)	28.56 (8.16)	0.268	0.097	29.28 (7.09)	29.12 (7.18)	0.022	0.909
**WA**	0.76 (1.36)	1.28 (2.70)	0.24	0.362	0.4 (0.57)	1.32 (2.06)	0.608	0.047	0.6 (1.08)	0.92 (3.09)	0.138	0.619
**NA**	27.08 (8.39)	30.96 (12.06)	0.373	0.037	30.36 (8.14)	27.24 (8.32)	0.379	0.020	28.80 (7.40)	28.20 (7.04)	0.083	0.593
**STAI-S**	35.48 (8.86)	31.56 (8.80)	0.44	0.012	35.76 (9.68)	30.24 (7.48)	0.638	0.003	35.96 (10.64)	29.7 6(9.19)	0.62	0.002
**Boys (*n* = 36)**												
**TA**	26.67 (6.95)	27.92 (8.83)	0.157	0.355	26.33 (8.19)	26.56 (9.0)	0.026	0.835	26.97 (7.40)	28.44 (9.76)	0.169	0.251
**WA**	0.47 (1.08)	0.5 (1.03)	0.028	0.905	0.47 (1.06)	0.67 (1.82)	0.134	0.455	0.389 (0.99)	0.80 (2.12)	0.248	0.289
**NA**	26.19 (6.58)	27.42 (8.82)	0.158	0.375	25.86 (8.08)	25.89 (8.83)	0.003	0.979	26.58 (7.40)	27.63 (9.40)	0.124	0.316
**STAI-S**	39.06 (11.41)	33.08 (10.97)	0.534	<0.001	38.11 (11.20)	33.97 (11.18)	0.369	0.004	37.11 (9.61)	33.53 (10.91)	0.348	0.002

Abbreviations: SLCT: six letter cancellation test; TA: Total attempted in SLCT; WA: Wrongly attempted in SLCT; NA; net attempted in SLCT; STAI-S: Spielberger’s state trait anxiety inventory; HFYB: High frequency yoga breathing; BAW: Breath awareness; QS: Quiet sitting.

**Table 3:** Details of repeated measures analysis of variance.

Whole Group (*n* = 61)							
Sn.	Variables	Sources	df	MS	F	*p*-Value	Partial Eta Squared
1	STAI	Sessions	2,120	16.167	0.585	0.559	0.010
		States	1,60	2135.126	39.606	<0.001	0.398
		Sessions × States	1893,113.580	2.203	0.103	0.892	0.002
2	TA	Sessions	2.000,120.000	15.257	0.415	0.661	0.007
		States	1,60	67.347	2.462	0.122	0.039
		Sessions × States	2.000,120.000	83.638	2.940	0.057	0.047
3	WA	Sessions	1.820,109.184	0.057	0.020	0.974	0.000
		States	1,60	12.265	3.563	0.064	0.056
		Sessions × States	1.807,108.396	0.584	0.262	0.748	0.004
4	NA	Sessions	1.991,119.486	15.882	0.414	0.661	0.007
		States	1,60	20.680	0.914	0.343	0.015
		Sessions × States	2,120	97.607	3.766	0.026	0.059
**Girls (*n* = 25)**							
1		Sessions	1.828,43.877	6.615	0.199	0.801	0.008
	STAI	States	1,24	1019.207	14.595	0.001	0.378
		Sessions × States	1.883,45.201	18.187	1.137	0.327	0.045
2		Sessions	1.872,44.931	9.451	0.191	0.813	0.008
	TA	States	1,24	17.340	0.457	0.505	0.019
		Sessions × States	2,48	142.740	5.791	0.006	0.194
3		Sessions	1.802,43.252	0.954	0.255	0.753	0.011
	WA	States	1,24	12.907	2.282	0.144	0.087
		Sessions × States	1.699,40.044	1.398	0.409	0.630	0.017
4		Sessions	1.795,43.081	3.796	0.066	0.920	0.003
	NA	States	1,24	0.107	0.004	0.951	0.000
		Sessions × States	2,48	157.127	6.817	0.002	0.221
**Boys (*n* = 36)**							
1		Sessions	2,70	13.019	0.492	0.614	0.014
	STAI	States	1,35	1125.227	25.420	<0.001	0.421
		Sessions × States	1.848,64.671	30.447	1.220	0.300	0.034
2		Sessions	2,70	29.866	0.977	0.381	0.027
	TA	States	1,35	52.019	2.498	0.123	0.067
		Sessions × States	1.957,68.491	8.181	0.267	0.762	0.008
3		Sessions	1.925,67.378	0.250	0.108	0.891	0.003
	WA	States	1,35	2.449	1.266	0.268	0.035
		Sessions × States	1.885,65.978	0.727	0.470	0.616	0.013
4		Sessions	1.981,69.337	30.132	1.014	0.368	0.028
	NA	States	1,35	31.894	1.615	0.212	0.044
		Sessions × States	2,70	7.532	0.282	0.755	0.008

Abbreviations: TA: total attempted in SLCT; WA: wrongly attempted in SLCT; NA; net attempted in SLCT; STAI-S: Spielberger’s state trait anxiety inventory.
